# Posttreatment of Domestic Wastewater Using Green Coconut Biochar for Coriander Fertigation

**DOI:** 10.1002/wer.70536

**Published:** 2026-08-24

**Authors:** Antonio Magno dos Santos Souza, Nicole Helen Lima Cardeal, Fabiano Santos Santana, Luciano de Melo, Gregório Guirado Faccioli, Fernando França da Cunha

**Affiliations:** ^1^ Department of Agricultural Engineering (DEA) Federal University of Viçosa (UFV) Viçosa Minas Gerais Brazil; ^2^ Federal Institute of Education, Science and Technology of Sergipe (IFS) Estância Sergipe Brazil; ^3^ Department of Agricultural Engineering (DEAGRI) Federal University of Sergipe (UFS) São Cristóvão Sergipe Brazil

**Keywords:** adsorption, biofilters, domestic effluents, filtration, wastewater treatment, water reuse

## Abstract

Increasing water scarcity and the need for sustainable agricultural intensification have encouraged the use of treated wastewater for fertigation. However, limitations in effluent quality still restrict its safe agricultural reuse. This study evaluated green coconut biochar as a posttreatment filter for domestic wastewater and its effects on coriander (*Coriandrum sativum L*.) performance. Treated wastewater, with and without biochar filtration, was applied at different proportions mixed with supply water over two cultivation cycles under protected conditions. The posttreatment increased dissolved oxygen and reduced thermotolerant coliforms by more than 99%, without significantly affecting salinity. Filtered wastewater also improved plant growth, biomass accumulation, and water use efficiency, particularly at intermediate wastewater proportions. Regression analyses suggested an expanded range of wastewater application associated with optimal crop performance. These findings suggest that green coconut biochar is a promising low‐cost alternative for domestic wastewater posttreatment, supporting improved effluent quality and safer agricultural reuse.

## Summary

- Biochar filtration improved wastewater quality for agricultural reuse.
- About 99% of thermotolerant coliforms were removed after posttreatment.
- The posttreatment increased dissolved oxygen without significant salinity changes.
- Posttreatment of domestic wastewater improved coriander growth and water productivity under fertigation.
- Filtered water maintained higher crop performance at intermediate wastewater proportions.

## Graphical abstract

Green coconut biochar improved the quality of treated domestic wastewater by reducing microbial contamination and increasing dissolved oxygen. The posttreated effluent supported coriander growth and water productivity, demonstrating its potential for safe agricultural reuse.

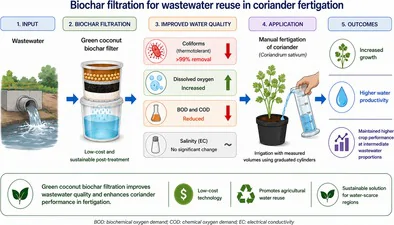

## 1 Introduction

Increasing water scarcity, combined with the intensification of food demand, has driven the adoption of more sustainable water‐use strategies in agriculture. In this context, the reuse of domestic wastewater (WW) in fertigation has emerged as a promising alternative, as it enables the simultaneous use of water and nutrients present in the effluent (Osorio et al. 2025). In addition to reducing pressure on conventional water sources, this practice may contribute to decreasing the use of mineral fertilizers and strengthening more resilient agricultural systems, especially in regions subject to water scarcity.

However, the direct use of treated effluents still presents important limitations related to the presence of pathogenic microorganisms, residual organic matter, and potentially phytotoxic compounds (Souza et al. 2022; Trotta et al. 2024). Furthermore, continuous application may promote salt accumulation in the soil, compromising crop development and the sustainability of the production system (Obijianya et al. 2025). Recent studies have also highlighted concerns regarding the presence of emerging contaminants and the cumulative effects of agricultural WW reuse on soil and water quality (Wang et al. 2024; Maldou et al. 2026; Otaru and Baba 2026). Therefore, the development of complementary technologies capable of promoting the posttreatment of these effluents in an efficient, accessible, and environmentally sustainable manner is required.

In this scenario, biochar has emerged as a promising material for application in water treatment systems due to its high surface area, porous structure, and adsorption capacity (Yu et al. 2019). In addition, the material has the potential to reduce turbidity, suspended solids, organic compounds, and part of the microbiological load present in domestic effluents (Alshehri and Pugazhendhi 2024; Bui et al. 2024). In particular, green coconut biochar presents additional advantages, such as wide availability, low cost, and high chemical stability, characteristics that favor its application in decentralized water treatment systems for agricultural reuse (Mishra et al. 2025). Its use may contribute to improving the physicochemical and microbiological quality of the effluent, as well as to the removal of undesirable compounds that limit crop agronomic performance.

Despite the growing interest in biochar‐based technologies for WW treatment, studies integrating biochar characterization, posttreatment performance, and agronomic evaluation under different treated WW application rates and successive cultivation cycles remain limited. In particular, little information is available regarding the use of green coconut biochar as a filtration medium for domestic WW intended for the production of leafy vegetables consumed fresh, where microbiological water quality is especially critical. Addressing this knowledge gap is essential to better understand the practical potential of green coconut biochar as a low‐cost technology for agricultural water reuse.

Coriander (*Coriandrum sativum L*.), owing to its short growing cycle and sensitivity to irrigation water quality (Hanif et al. 2024; Navarro et al. 2024), provides an appropriate model crop for evaluating these effects. We hypothesized that posttreatment of domestic WW using green coconut biochar would improve the physicochemical and microbiological quality of the effluent, thereby enhancing coriander growth and water productivity compared with conventionally treated WW, while maintaining the nutrient supply required for crop development.

Given the above, this study aimed to evaluate the potential of green coconut biochar as a filtering material in the posttreatment of domestic WW and to investigate its effects on water quality and the agronomic performance of coriander, considering different application proportions and successive cultivation cycles.

## 2 Materials and Methods

### 2.1 Biochar Production

The biochar was produced from whole green coconuts ( *Cocos nucifera* L., Typica variety) collected locally. Only the coconut water was removed prior to fragmentation and pyrolysis. The material was fragmented, air‐dried under ambient conditions, and subsequently oven‐dried at 80°C for 24 h. After drying, the material was ground and sieved to obtain particles with a size of up to 3 mm and then homogenized.

Carbonization was carried out in a muffle furnace. The samples were placed in porcelain crucibles and subjected to a temperature of 550°C, with a heating rate of 25°C min^−1^, remaining under these conditions for 60 min. After the process, the biochar was cooled to room temperature, homogenized, and stored for subsequent use.

The choice of carbonization temperature was based on previous studies indicating improved physicochemical properties of the material under this condition. To perform the characterization analyses and assemble the filtration system, the procedure was repeated until a sufficient quantity of biochar was obtained, and all the material was subsequently homogenized.

### 2.2 Biochar Characterization

The physicochemical characterization of the biochar was performed before and after its use as a filtering material. The analyses were conducted at the Multiuser Chemistry Laboratory Center of the Federal University of Sergipe (UFS).

Thermal properties were evaluated by thermogravimetric analysis (TGA) using a Shimadzu instrument (model TG‐50A), within the temperature range of 30°C–1000°C, under a nitrogen atmosphere and a heating rate of 10°C min^−1^.

Elemental composition (C, H, and N) was determined using a CHN analyzer (LECO CHN628). The instrument was operated with helium (99.995% purity) and oxygen (99.999% purity), with furnace temperature set at 950°C and afterburner temperature at 850°C. Other parameters were adjusted to improve sensitivity. The instrument was calibrated using an EDTA standard (41.0% C, 5.5% H, and 9.5% N), with a mass range from 10 to 200 mg. Approximately 50 mg of each sample was weighed in tin foil for the analyses.

Chemical composition was determined by energy‐dispersive X‐ray fluorescence spectrometry (EDXRF) using a Shimadzu instrument (model EDX‐720). The X‐ray tube voltage was set at 15 and 50 keV, with a current of 100 μA, detector dead time of 40%, and a 10‐mm collimator. Spectra were sequentially obtained from 0 to 40 keV. The irradiation time was 100 s.

Surface morphology was analyzed by scanning electron microscopy (SEM) using a Hitachi microscope (model TM3000), with previously metallized samples. The instrument was operated at 15 kV, and image magnification ranged from 50 to 10,000×.

Specific surface area, pore volume, and pore size distribution were determined using the BET method with a Quantachrome instrument (model NOVA 1200e), after sample degassing at 200°C. The analysis was conducted with an equilibrium time of 60 s for each adsorption and desorption point, pressure tolerance of 0.100, and maximum stabilization time of 240 s. The dry weight of each sample was approximately 100 mg.

### 2.3 Experimental Assay

The experiment was conducted in a greenhouse under bench‐scale conditions using 6‐L cylindrical polyethylene pots, approximately 22 cm high and 21 cm in diameter, provided with perforations at the base for drainage.

Two successive cultivation cycles of coriander ( *C. sativum* L.) were conducted. The first cycle began on September 8, 2024, with harvest performed on October 18, 2024. The second cycle was sown on October 28, 2024, and harvested on December 6, 2024. These cycles were carried out at the Department of Agronomic Engineering of the Federal UFS, located in São Cristóvão, Sergipe, Brazil.

The pots were filled with previously prepared soil and arranged to ensure experimental uniformity. The experiment was conducted under manual irrigation using different water sources according to the established treatments.

Water management was based on daily crop evapotranspiration (ETc) replacement, aiming to maintain soil moisture close to field capacity throughout the cultivation cycle. The water sources used in the experiment were obtained from bench‐scale filtration systems, as described below.

### 2.4 Filtration System

**FIGURE 1:**
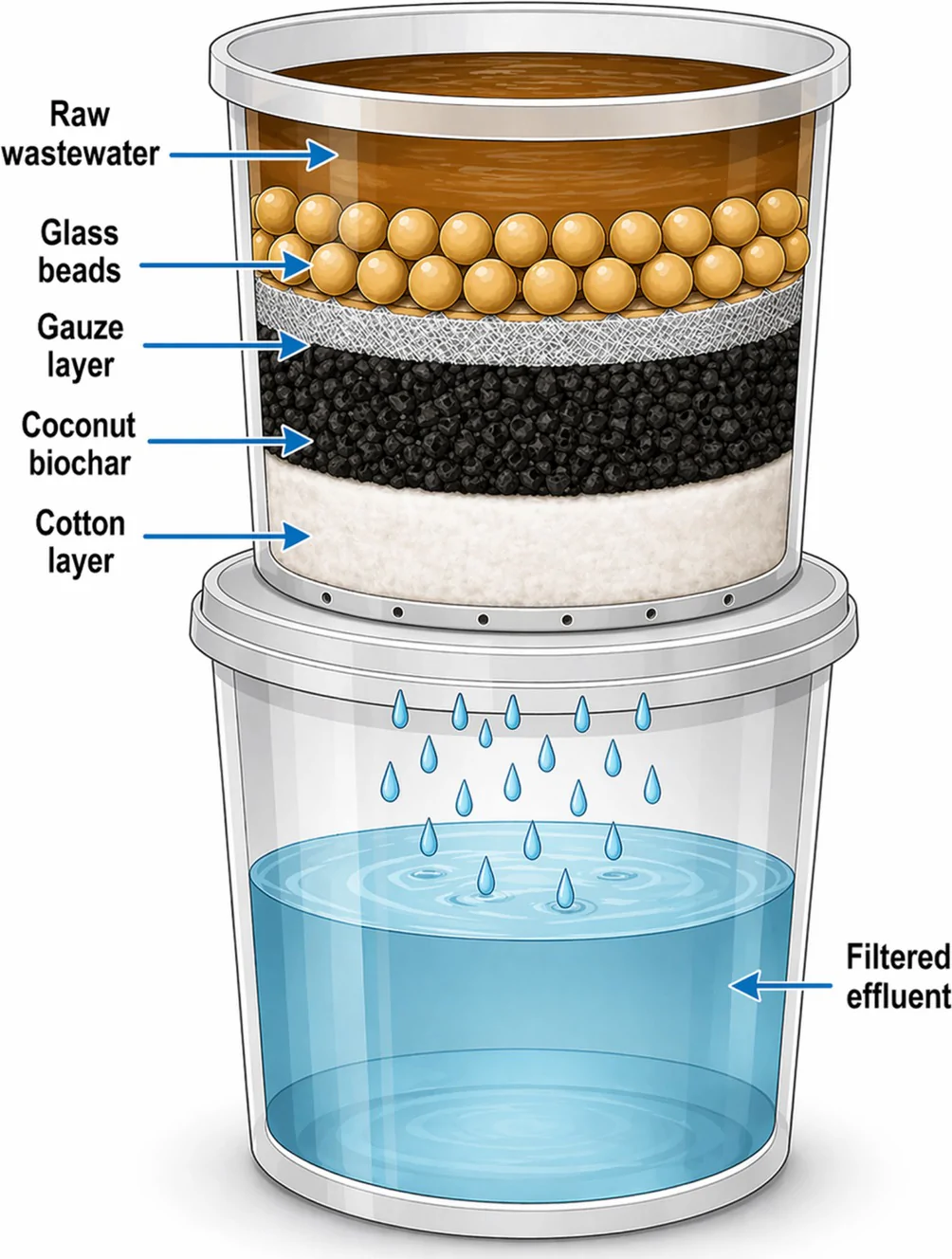
Schematic illustration of the filtration system.

The filtration systems were constructed at bench scale using vertically arranged 5‐L plastic containers as columns (Figure 1).

At the base of each column, a 5‐cm cotton layer was added to support the filtering material. Above this layer, a 5‐cm layer of green coconut biochar was added, acting as the adsorptive medium. To prevent material dispersion during operation, a gauze layer was placed over the biochar. In the upper part of the column, a 15‐cm layer of glass beads was added to dissipate flow energy and promote uniform distribution of the effluent.

The treated domestic WW was applied at the top of the columns, percolating through the filtering layers, and the filtrate was collected at the base of the system for subsequent use in fertigation.

For filtered effluent collection, 20‐L plastic containers were used, adapted with lids containing openings compatible with the column diameter, allowing proper fitting of the system.

### 2.5 Water Sources

Three water sources were used for fertigation: (i) potable water supplied by the local water utility and collected inside the protected environment; (ii) treated domestic WW obtained from a WW treatment plant located in Estância, Sergipe State, Brazil; and (iii) WW posttreated through a filtration system containing green coconut biochar.

The different proportions between WW and potable water were established according to the experimental design.

### 2.6 Experimental Design

The experiment was conducted in a randomized block design with four replicates. A 2 × 5 + control factorial scheme was adopted.

The evaluated factors consisted of two WW sources: (i) treated domestic WW and (ii) treated domestic WW posttreated by filtration with green coconut biochar. Each WW source was independently diluted with potable water to obtain irrigation solutions containing 20%, 40%, 60%, 80%, and 100% WW, resulting in 10 treatments. An additional control treatment consisted exclusively of potable water and received fertilization according to crop recommendations.

Each experimental unit consisted of one pot containing one plant, totaling 44 experimental units. Treatments were randomly distributed within each block.

### 2.7 Soil

The soil used in the experiment was collected from the 0‐ to 20‐cm layer at the Rural Campus of the Federal UFS. The soil was subsequently sieved, homogenized, and placed into pots over a gravel layer intended for drainage. The soil physicochemical characteristics are presented in Table 1.

**TABLE 1:** Chemical and physical characterization of the soil (0–20 cm) before the beginning of the experiment.

Parameter	Value	Unit
pH in water	5.83	—
Phosphorus (P)	2.00	mg dm^−3^
Potassium (K)	23.70	mg dm^−3^
Sodium (Na)	4.70	mg dm^−3^
Organic matter (OM)	3.22	g dm^−3^
Calcium (Ca^2+^)	0.58	cmol_c_ dm^−3^
Magnesium (Mg^2+^)	0.35	cmol_c_ dm^−3^
Aluminum (Al^3+^)	< 0.08	cmol_c_ dm^−3^
Hydrogen + Aluminum (H + Al)	1.24	cmol_c_ dm^−3^
Sum of exchangeable bases (SB)	1.01	cmol_c_ dm^−3^
Cation exchange capacity (CEC)	2.25	cmol_c_ dm^−3^
Base saturation (V)	44.90	%
Exchangeable sodium percentage (ESP)	0.89	%
Electrical conductivity (25°C)	0.35	dS m^−1^
Bulk density	1.37	g cm^−3^
Sand	86.14	%
Clay	7.36	%
Silt	6.50	%
Textural class	Sandy loam	—

The soil exhibited slightly acidic pH, low base saturation, low phosphorus content, low cation retention capacity, and low cation exchange capacity (CEC), characteristics consistent with its sandy texture.

Based on these attributes, soil acidity was corrected through lime application, followed by basal fertilization. Topdressing fertilization was performed only in the control treatment, according to crop recommendations.

### 2.8 Climatic Conditions

**FIGURE 2:**
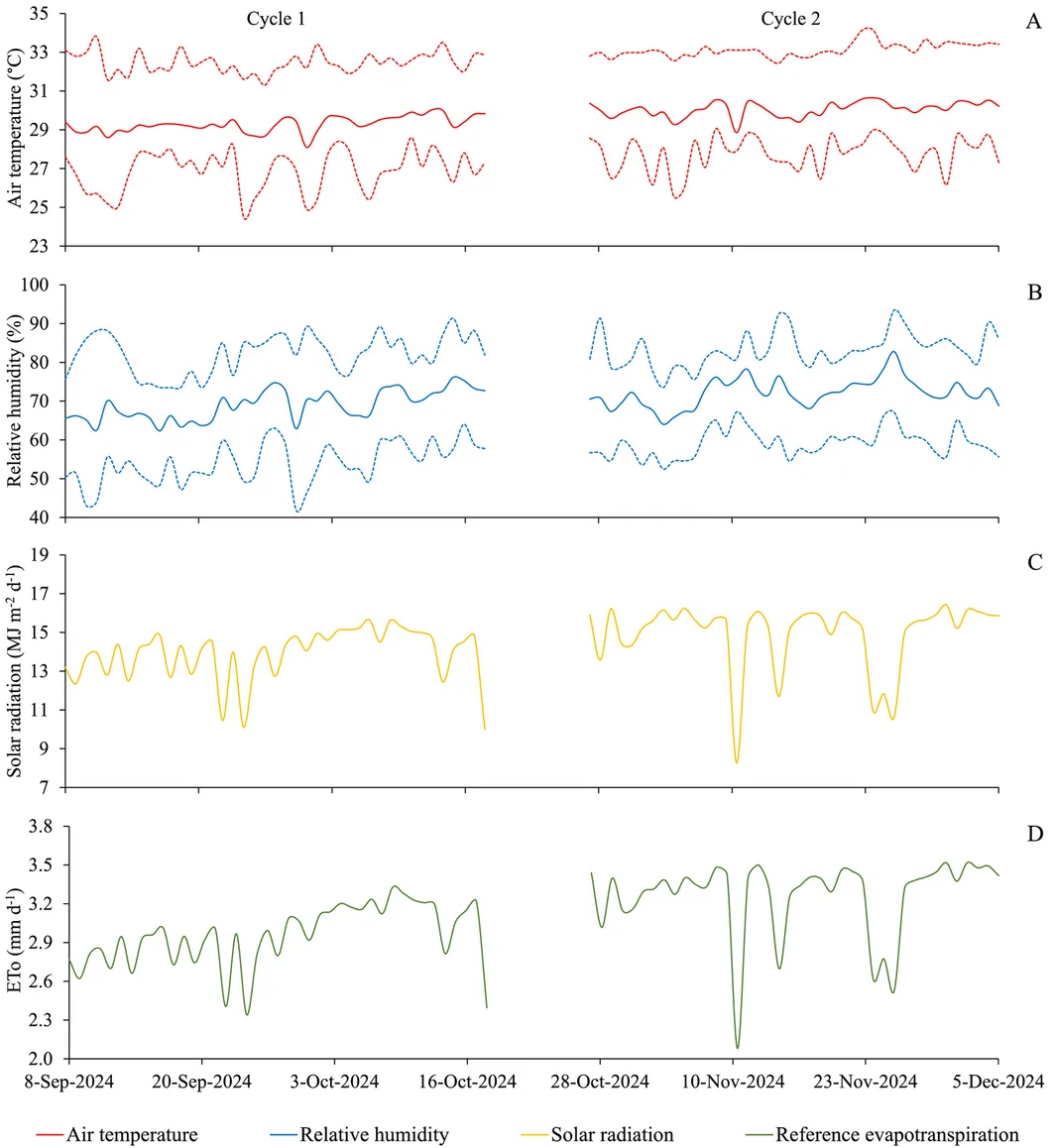
Daily maximum, mean, and minimum air temperature values (A) and relative humidity (B), as well as daily accumulated solar radiation (C) and daily reference evapotranspiration (D) during the experimental period.

Daily mean air temperature and relative humidity were calculated from all measurements recorded throughout each day, whereas minimum and maximum values corresponded to the extremes observed during the same period. Daily solar radiation was obtained by integrating records collected every 5 min over a 24‐h period. Based on these variables, reference ETc was estimated using the Penman–Monteith method (Pereira et al. 2025). The daily variations in air temperature, relative humidity, solar radiation, and reference ETc are presented in Figure 2.

During the first cultivation cycle, daily solar radiation ranged from 10.0 to 15.7 MJ m^−2^ day^−1^, with a mean of 13.9 MJ m^−2^ day^−1^, whereas reference ETc ranged from 2.34 to 3.33 mm day^−1^, with a mean of 2.95 mm day^−1^. During the second cycle, daily solar radiation ranged from 8.3 to 16.4 MJ m^−2^ day^−1^ (mean of 14.9 MJ m^−2^ day^−1^), while reference ETc ranged from 2.08 to 3.52 mm day^−1^, with a mean of 3.27 mm day^−1^. The complete daily records of air temperature, relative humidity, solar radiation, and reference ETc are shown in Figure 2.

### 2.9 Coriander Cultivation and Management

Coriander, cv. Verdão, was directly sown into 6‐L pots using five seeds per experimental unit. After seedling emergence, thinning was performed to ensure experimental uniformity, maintaining one plant per pot.

During the first 7 days after sowing, all pots were irrigated with potable water to standardize initial plant establishment. After this period, the irrigation treatments were applied according to the experimental design.

Basal fertilization was performed at sowing in all pots. No chemical control of pests or diseases was required throughout the cultivation cycle.

### 2.10 Water Management and Estimation of Crop ETc

Water management was based on daily replacement of crop ETc, estimated by the direct pot‐weighing method. Daily, the mass of each experimental unit was determined, and ETc was calculated based on the difference between pot mass at field capacity and pot mass at the time of evaluation, with correction for plant growth throughout the cycle. ETc was estimated according to Equation (1):

(1) ETc=FC−CM+Pi

where ETc is crop evapotranspiration (L pot^−1^), FC is the pot mass at field capacity (kg), CM is the pot mass at current moisture content (kg), and Pi is the plant mass on the evaluation day (kg).

To estimate plant mass variation throughout the cycle, additional plants were cultivated under the same experimental conditions. Daily, one plant was collected and weighed, and this value was used to correct the equation.

Irrigation was performed manually by applying the water volume corresponding to the estimated ETc for each treatment, aiming to restore soil moisture close to field capacity. Water was applied directly to the soil in each pot using graduated containers to ensure application accuracy.

### 2.11 Analysis of Water Sources

Water quality was evaluated through physical, chemical, and microbiological analyses. The following physical and chemical parameters were determined: pH, electrical conductivity (EC), biochemical oxygen demand (BOD), chemical oxygen demand (COD), dissolved oxygen (DO), and total phosphorus. Microbiological evaluation was performed by quantifying thermotolerant coliforms. Samples were collected every two weeks throughout the cropping cycle, stored in appropriate containers, and sent to a certified laboratory for analysis.

In addition to the laboratory analyses, pH, EC, temperature, and salinity were monitored daily using a portable multiparameter meter to continuously assess the quality of the irrigation water throughout the experiment. These measurements were used to evaluate the performance of the filtration system and the feasibility of treated WW reuse for fertigation.

### 2.12 Analysis of Coriander Agronomic Characteristics

At the end of each cultivation cycle, the following agronomic variables were evaluated: plant height, shoot fresh mass (SFM), shoot dry mass (SDM), and water productivity. Plant height was measured using a millimeter ruler from the soil surface to the tip of the tallest leaf at harvest.

After sampling, the shoots were separated from the root system using pruning scissors. Subsequently, shoot samples were placed in properly identified paper bags and weighed to determine fresh mass using a precision electronic balance (0.01 g). The samples were then dried in a forced‐air circulation oven at 65°C for 72 h until constant mass was achieved. Subsequently, SDM was determined using a precision electronic balance.

Water productivity (WP) was calculated as the ratio between SFM and the total volume of water consumed during the cultivation cycle, with results expressed in kg m^−3^.

### 2.13 Statistical Analysis

Agronomic variables were initially evaluated for compliance with the assumptions of analysis of variance (ANOVA). Residual normality was assessed using the Shapiro–Wilk test, and homogeneity of variances was evaluated using Bartlett's test, both at the 5% significance level. Subsequently, the variables were subjected to analysis of variance (ANOVA), considering a randomized block design at the 5% significance level using the F‐test.

To better understand the behavior of the variables as a function of the evaluated factors, interaction effects were further analyzed regardless of their significance in the ANOVA through simple effect analyses.

Comparison between the control and the other treatments was performed using Dunnett's test (*p* ≤ 0.05), whereas means among water sources were compared using Tukey's test (p ≤ 0.05). For quantitative factors, regression models were fitted and selected based on coefficient significance, coefficient of determination (R^2^), and biological consistency.

Statistical analyses were performed using R software (version 3.6.3), and regression graphs were generated using SigmaPlot software (version 15.0).

## 3 Results and Discussion

### 3.1 Biochar Characterization

#### 3.1.1 TGA (TG/DTG)

**FIGURE 3:**
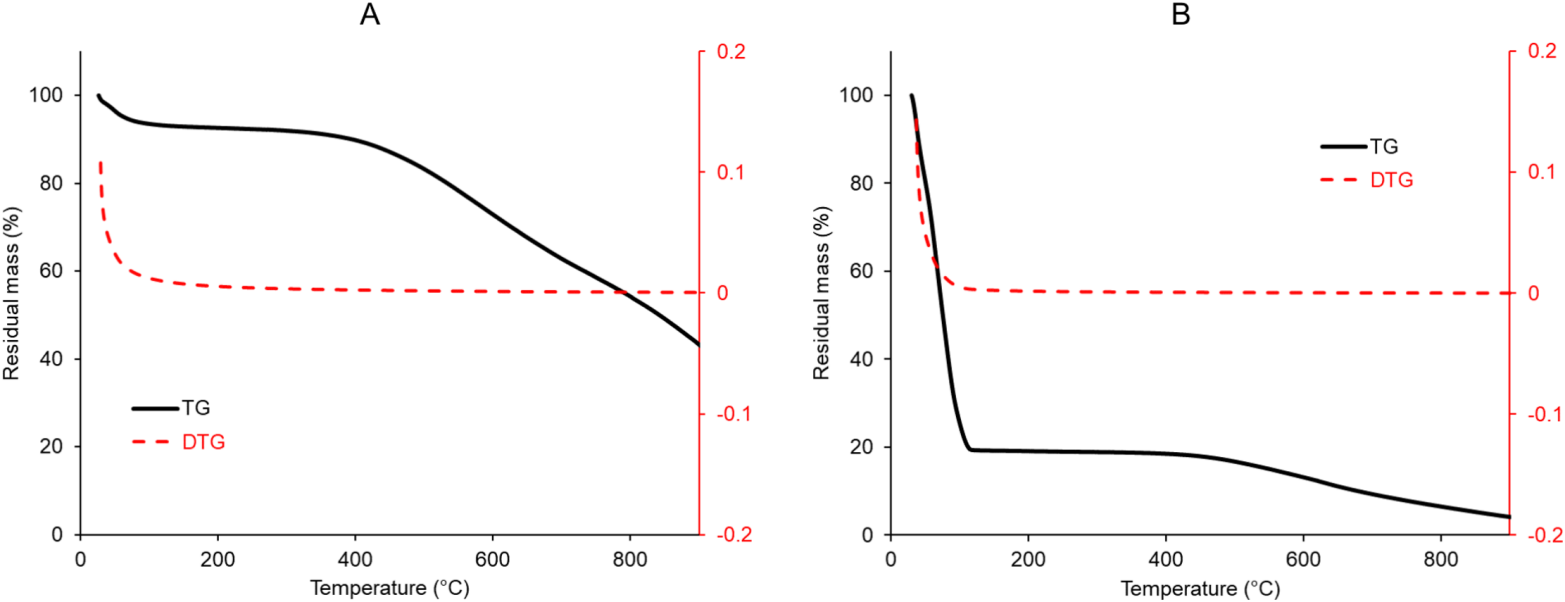
Thermogravimetric curves of green coconut biochar: (A) pristine biochar; (B) biochar after wastewater filtration.

The thermogravimetric (TG) and derivative thermogravimetric (DTG) curves of green coconut biochar, in both fresh and postfiltration conditions, are presented in Figure 3. Differences in thermal behavior were observed as a function of the material used in effluent treatment.

At lower temperature ranges (from 30°C to 143°C), the green coconut biochar used for WW filtration exhibited an initial mass loss of 7.026 mg (76.39%). In contrast, within a similar temperature range (26°C to 100°C), pristine green coconut biochar showed a lower mass loss of 0.381 mg (5.84%). This difference suggests that the biochar used in WW filtration retained higher amounts of volatile substances, water, and possibly organic compounds.

At intermediate temperature ranges (from 365°C to 865°C for the postfiltration biochar and from 361°C to 661°C for the pristine biochar), mass losses of 1.267 mg (13.78%) and 1.593 mg (24.40%) were observed, respectively. This degradation may be attributed to the decomposition of the carbonaceous fraction of the material, particularly residual cellulose and lignin. Similar results were reported by Hanif et al. (2023), who observed substantial mass loss between 300°C and 450°C, associated with hemicellulose and cellulose degradation.

In the final temperature range, between 818°C and 996°C, only the pristine green coconut biochar exhibited a considerable mass loss (1.337 mg, corresponding to 20.48%), whereas the postfiltration material showed greater thermal stability, with no significant mass loss at this stage. This behavior corroborates the findings of Pang et al. (2021), who reported the onset of decomposition above 350°C and a final residue content of 42% at 1000°C, indicating high thermal resistance of green coconut biochar.

#### 3.1.2 Elemental Composition (CHN)

**FIGURE 4:**
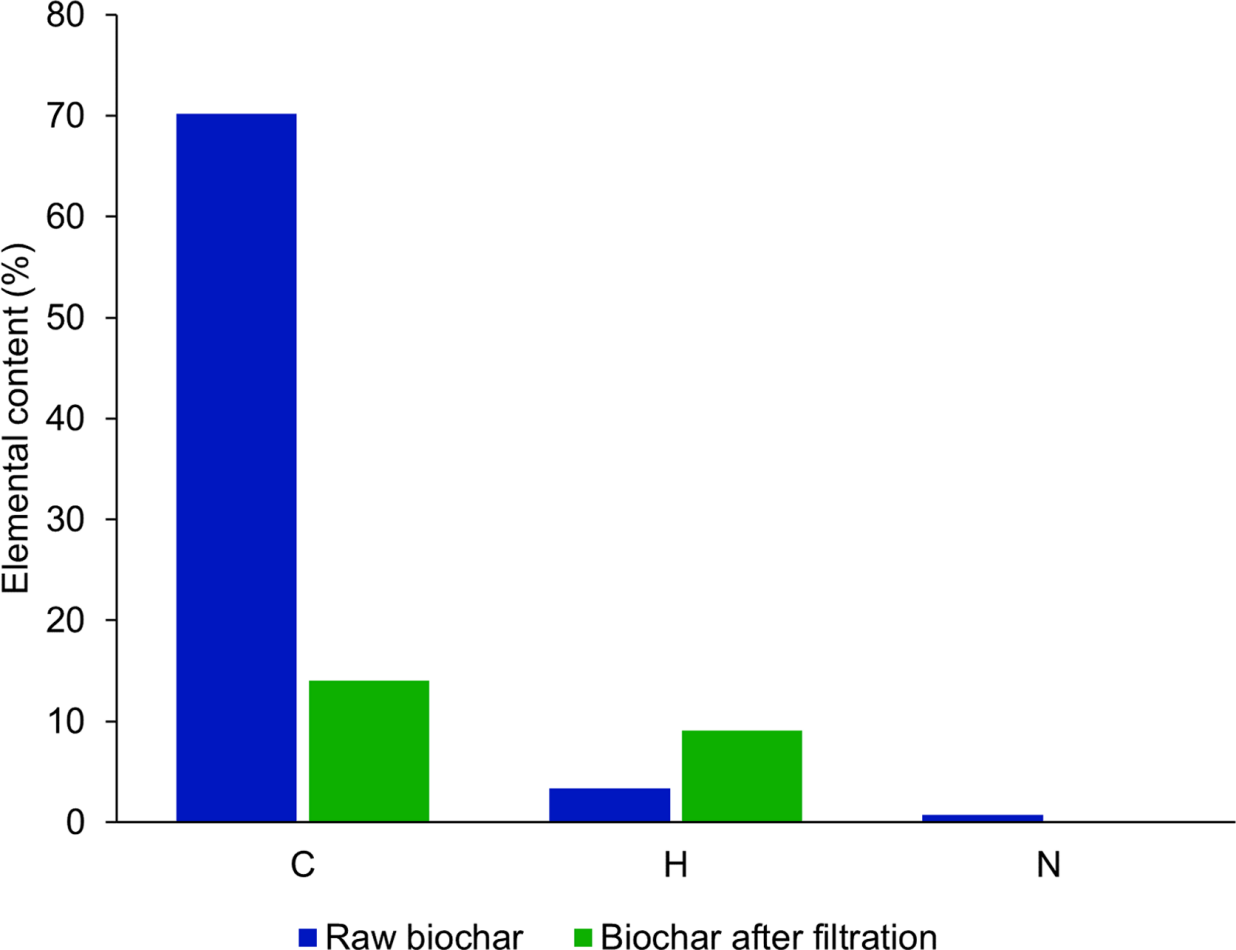
Elemental contents of carbon (C), hydrogen (H), and nitrogen (N) in green coconut biochar before and after domestic wastewater filtration.

The elemental composition of green coconut biochar revealed changes in carbon (C), hydrogen (H), and nitrogen (N) contents after its use as a filtration medium (Figure 4).

Pristine biochar exhibited a high carbon content (70.22%), a characteristic typically observed in materials subjected to pyrolysis at elevated temperatures, reflecting a higher degree of carbonization and greater fixed carbon content. This behavior is associated with the high lignin content of the original biomass, whose compounds exhibit greater resistance to thermal degradation. Studies by Rizzioli et al. (2024), Salam et al. (2022), and El‐Gamal et al. (2017) reported that biochars produced at 550°C tend to exhibit high carbon contents due to the concentration of fixed carbon following the thermal degradation of organic matter.

After the WW filtration process, a substantial reduction in carbon content was observed, decreasing from 70.22% to 14.06%. This reduction may be associated with the incorporation of compounds from the WW, rich in oxygen, hydrogen, and other elements, as well as the deposition of organic matter on the biochar surface, contributing to the relative dilution of carbon in the elemental composition. According to Qiu et al. (2023), contact between biochar and WW may promote surface modifications in the material, altering its chemical composition.

Hydrogen content increased after the use of the material as a filtration medium, rising from 3.34% to 9.05%. This behavior indicates greater retention of organic compounds and moisture, demonstrating the interaction between the material and substances present in the WW. Similar results were reported by Poopisut et al. (2023), who observed that biochars produced at 550°C were capable of reabsorbing moisture when exposed to the environment, thereby influencing their elemental composition.

Regarding nitrogen, a reduction in content was observed after the filtration process, decreasing from 0.70% to 0.14%. This behavior may be associated with the leaching of more soluble nitrogenous compounds during WW percolation or with chemical transformations of these compounds throughout the filtration process. Furthermore, the incorporation of other elements into the biochar matrix may have contributed to the relative reduction in nitrogen content.

Overall, the observed changes in elemental composition indicate that green coconut biochar undergoes significant modifications after its use as a filtration medium, reflecting its ability to interact with compounds present in domestic WW.

#### 3.1.3 Energy‐Dispersive X‐Ray Fluorescence (EDX)

The elemental composition of green coconut biochar determined by energy‐dispersive X‐ray fluorescence (EDX), both in the pristine condition and after use as a filtration medium for domestic WW, is presented in Table 2. The biochar used for postfiltration characterization was collected from the filtration column after completion of the second cropping cycle. The results revealed substantial changes in the chemical composition of the material after the filtration process.

**TABLE 2:** Elemental composition (%) determined by energy‐dispersive X‐ray fluorescence (EDX) of pristine green coconut biochar and biochar after use as a filtration medium for domestic wastewater.

Parameter	Pristine green coconut biochar (%)	Green coconut biochar after wastewater filtration (%)
K	68.420	4.650
Cl	23.273	—
Ca	5.726	70.079
P	1.183	—
Fe	0.620	13.978
S	0.396	—
Zn	0.152	6.921
Cu	0.071	4.373
Br	0.061	—
Ag	0.056	—
Rb	0.027	—
Sr	0.015	—

*Note:* — concentration below the detection limit.

A reduction in potassium content was observed after WW filtration, decreasing from 68.42% to 4.65%, while elements such as chlorine, phosphorus, and sulfur were no longer detected. This behavior indicates the possible desorption or solubilization of these ions during the filtration process, particularly those with greater mobility in aqueous media.

In contrast, the substantial increase in calcium content (from 5.73% to 70.08%), as well as in iron, zinc, and copper concentrations in the green coconut biochar, indicates an efficient adsorption process of these elements present in domestic WW. Similar accumulation was reported by Mian et al. (2024), who used EDX analysis to demonstrate the retention of Ca, Fe, Zn, and Cu in biochars applied to agro‐industrial WW treatment. Likewise, Khan et al. (2024) attributed this retention to mineral accumulation and the presence of functional groups in the carbonaceous matrix. Sahoo et al. (2023) also observed changes in the surface composition of adsorbent materials after contact with WW, with mineral accumulation detected by EDX, corroborating the processes identified in the present study.

#### 3.1.4 SEM

**FIGURE 5:**
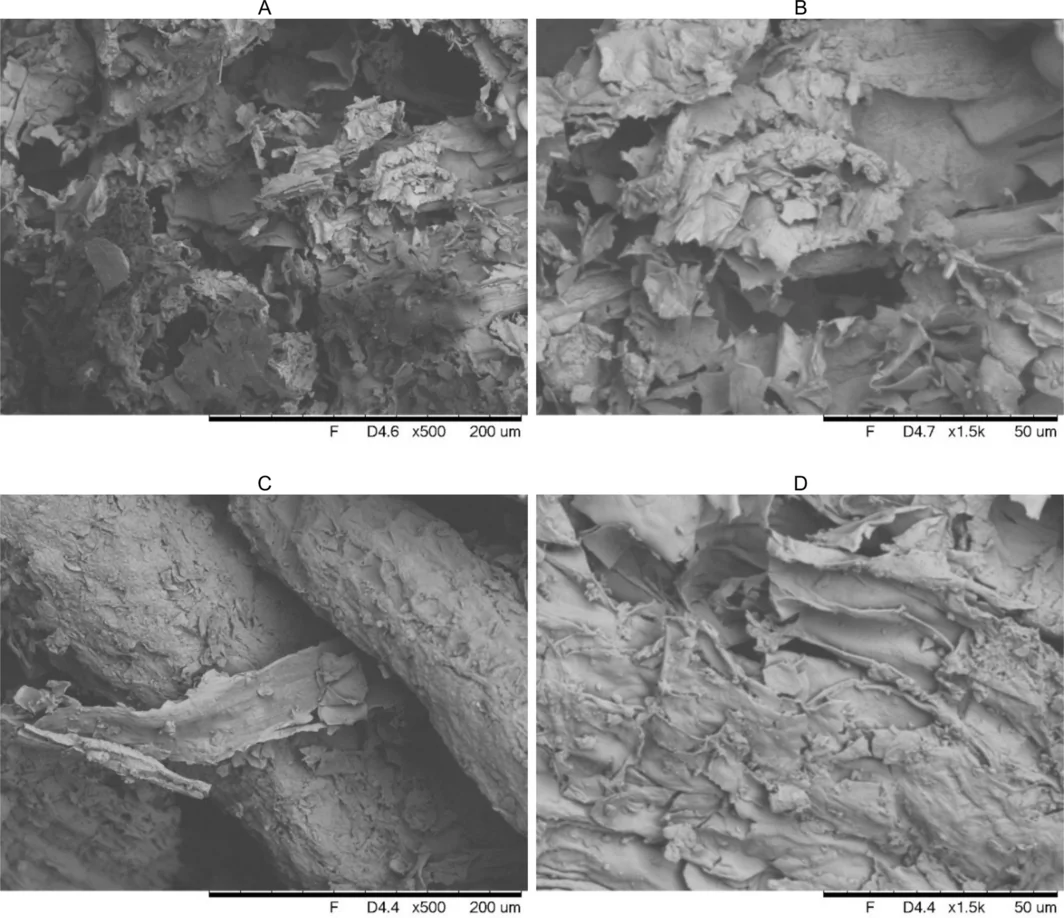
Scanning electron microscopy micrographs of green coconut biochar before and after wastewater filtration under different conditions: (A) pristine biochar—500×; (B) pristine biochar—1500×; (C) biochar after wastewater filtration—500×; (D) biochar after wastewater filtration—1500×.

SEM was used to evaluate the morphology of green coconut biochar in its pristine condition and after its use as a filtration medium for domestic WW. Representative SEM micrographs of pristine and WW‐filtered green coconut biochar at different magnifications are shown in Figure 5.

Pristine biochar exhibited a highly porous structure, with interconnected cavities and well‐defined walls, which are characteristic of carbonized lignocellulosic materials. This structural configuration favors high specific surface area and the availability of active sites for adsorption processes.

After its use as a filtration medium, changes in the surface morphology of the material were observed. The micrographs revealed the presence of material adhered to the surface, as well as regions with reduced cavity definition. At higher magnifications, partial pore filling was observed, indicating the deposition of particles and substances derived from the WW.

These modifications suggest the retention of organic matter and suspended solids throughout the filtration process, corroborating the results obtained from the chemical and elemental analyses. It should be emphasized that SEM analysis is qualitative *in nature*, and therefore, the degree of pore obstruction cannot be quantified solely based on the micrographs.

#### 3.1.5 Textural Parameters and Adsorption Isotherms

The textural parameters of green coconut biochar obtained from BET and BJH analyses are presented in Table 3.

**TABLE 3:** Textural parameters of green coconut biochars.

Sample	BET surface area (m^2^ g^−1^)	Specific pore volume^a^ (cm^3^ g^−1^)	Average pore radius^b^ (nm) (ads./des.)
Pristine green coconut biochar	20.66	0.00643	1.823/7.807
Green coconut biochar after wastewater filtration	65.77	0.01513	1.798/1.794

^a^ Specific pore volume obtained by the cumulative BJH adsorption method.

^b^ Pore radius determined by the BJH method (adsorption/desorption), expressed in nanometers (nm).

The raw green coconut biochar presented a surface area of 20.66 m^2^ g^−1^, pore volume of 0.00643 cm^3^ g^−1^, and mean pore radius of approximately 1.82 nm (adsorption). After its use as a filtering material for domestic WW, a substantial increase in surface area was observed, reaching 65.77 m^2^ g^−1^, along with an increase in pore volume to 0.01513 cm^3^ g^−1^, while the mean pore radius remained practically unchanged.

The increase in BET surface area observed after filtration was unexpected, since pore blockage by retained particles would generally be anticipated. One possible explanation is the removal of residual pyrolytic compounds or the exposure of previously inaccessible pores during WW percolation. However, alternative explanations, including analytical variability, differences in sample preparation or drying, and the inherent heterogeneity of biochar, cannot be ruled out. Therefore, these results should be interpreted with caution and require further investigation. The mean pore radius values, ranging approximately from 1.8 to 7.8 nm, indicate the predominance of micropores to mesopores, a characteristic favorable for the adsorption of compounds present in WW.

**FIGURE 6:**
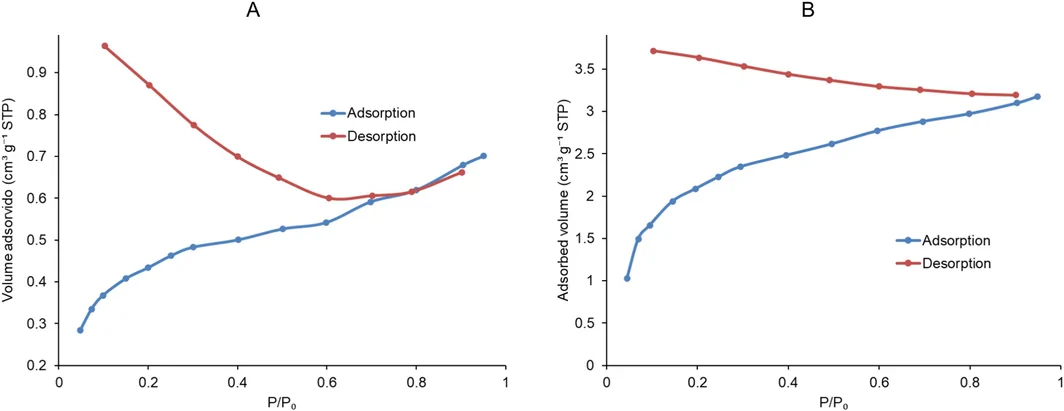
N_2_ adsorption and desorption isotherms of green coconut biochar: (A) pristine biochar; (B) biochar after wastewater filtration.

The N_2_ adsorption and desorption isotherms of the green coconut biochar are presented in Figure 6 for both the raw condition and after use in WW treatment.

The adsorption isotherms exhibited a typical type IV pattern with H4 hysteresis, according to the IUPAC classification (Sing et al. 1985), indicating a predominantly mesoporous structure with slit‐shaped pores. After WW filtration, an increase in the adsorbed volume was observed, corroborating the increase in surface area and adsorptive capacity of the material.

This behavior has been associated with the interaction between the biochar and the liquid medium, which may promote changes in the porous structure and increase the accessibility of active sites. Thus, the results indicate that green coconut biochar maintained its structure after use while exhibiting improved textural properties, reinforcing its potential application in WW posttreatment systems.

### 3.2 Filtration System and Water Quality for Fertigation

#### 3.2.1 Characterization of the Water Sources Used for Fertigation

The quality of the water sources used for fertigation was evaluated through physicochemical and microbiological parameters, considering the domestic WW from the WW treatment plant (WWTP) and the WW after filtration through green coconut biochar. A summary of the main results is presented in Table 4, with values expressed as the minimum and maximum ranges observed throughout the experimental period.

**TABLE 4:** Minimum and maximum values of the physicochemical and microbiological parameters of domestic wastewater before and after filtration through green coconut biochar during the experimental period.

Parameter	WWTP effluent	Biochar‐filtered effluent
BOD (mg L^−1^)	6.11–26.67	7.78–22.22
COD (mg L^−1^)	11–48	14–40
DO (mg L^−1^)	1.19–6.14	5.32–6.02
Phosphorus (mg L^−1^)	4.98–5.56	5.13–9.90
Coliforms (MPN 100 mL^−1^)	49–2200	4.0–49.0
pH	8.12–8.81	8.10–8.80
EC (mS cm^−1^)	1.196–1.242	1.221–4.12*
Salinity (g L^−1^)	0.69–0.79	0.66–2.25*
Temperature (°C)	26.4–29.9	26.2–29.5

*Note:* The highest EC and salinity values were observed immediately after a single replacement of the biochar filtering material during the first cropping cycle.

Abbreviation: MPN, most probable number.

Overall, the green coconut biochar filtration system promoted relevant improvements in effluent quality, particularly regarding oxygenation, reduction of organic load, and high microbiological efficiency.

BOD values ranged from 6.11 to 26.67 mg L^−1^ in the raw effluent and from 7.78 to 22.22 mg L^−1^ after filtration, suggesting a possible reduction in the biodegradable fraction of organic matter, with punctual reductions of up to 16.7%. Similarly, COD decreased from 11–48 mg L^−1^ in the WWTP effluent to 14–40 mg L^−1^ in the filtered effluent. These results may indicate that the biochar contributed to reducing the total organic load, especially after stabilization of the filtration system.

Regarding DO, a substantial increase was observed after filtration, with values ranging from 1.19 to 6.14 mg L^−1^ in the raw effluent to 5.32–6.02 mg L^−1^ in the treated effluent. This increase, exceeding 250% in some sampling events, suggests an improvement in water oxygenation conditions, reflecting lower oxygen consumption by organic matter and greater biological balance within the system. Although the mechanisms responsible for this increase were not directly evaluated, the reduction in oxygen demand associated with partial removal of suspended or biodegradable compounds may have contributed to this behavior.

Total phosphorus concentrations remained high after filtration, ranging from 4.98 to 5.56 mg L^−1^ in the WWTP effluent to 5.13–9.90 mg L^−1^ in the filtered effluent. The punctual increase observed may be associated with the release of previously adsorbed phosphorus or saturation of the active sites of the biochar. Nevertheless, the maintenance of this nutrient in the effluent may be advantageous for fertigation purposes, provided it is properly managed to avoid leaching losses or environmental impacts.

Microbiological analysis demonstrated high efficiency of the filtration system, with thermotolerant coliform reductions from 49–2200 MPN 100 mL^−1^ to 4.0–49.0 MPN 100 mL^−1^, corresponding to removal efficiencies greater than 99%. This behavior may be attributed to the porous structure of the biochar, which favors the physical retention of microorganisms, in addition to possible electrostatic interactions between the material surface and bacterial cells.

The physicochemical parameters related to salinity showed little variation after filtration. The pH remained within the range of 8.10 to 8.80, characterizing a slightly alkaline medium, with no significant differences between the raw and filtered effluents. EC ranged from 1.196 to 1.242 mS cm^−1^ in the WWTP effluent and from 1.221 to 4.12 mS cm^−1^ in the filtered effluent, while salinity ranged from 0.69 to 0.79 g L^−1^ and 0.66 to 2.25 g L^−1^, respectively. The higher values observed in the filtration system were associated with a single replacement of the biochar filtering material during the first cropping cycle. The replacement was performed because progressive saturation of the biochar reduced the filtration rate, resulting in a substantially slower flow through the column. The temporary increase in EC and salinity observed immediately after replacement likely reflects the initial release of soluble salts from the fresh biochar. In general, these results suggest that the biochar did not promote significant removal of dissolved salts.

Although the filtration system did not consistently reduce effluent salinity, substantial improvements were observed in microbiological quality and dissolved oxygen, factors that may have contributed to the improved agronomic performance observed in some treatments.

Water temperature ranged from 26.2°C to 29.9°C under all evaluated conditions, remaining within the suitable range for agricultural use and showing no significant influence from the filtration process.

Overall, the results indicate that green coconut biochar acts as an efficient posttreatment system, promoting improvements in sanitary quality and water oxygenation without compromising the availability of essential nutrients for crop development.

#### 3.2.2 Agronomic Characteristics of Coriander

Table 5 presents the mean squares, *F*‐test significance levels, and mean values obtained for all agronomic parameters analyzed. These results allow the identification of the main effects and interactions among water sources, WW proportions, and cultivation cycles.

**TABLE 5:** Mean squares, *F*‐test significance, and mean values of plant height, shoot fresh mass, shoot dry mass, and water productivity of coriander under different cultivation cycles, comparing three water sources: supply water (Control), conventionally treated wastewater (Treated), and treated wastewater filtered through a green coconut biochar biofilter (Filtered), both applied at different proportions with supply water.

Cultivation cycles			Cycle 1		Cycle 2	
Plant height (cm)	Mean square	WS	6.86**		4.85 × 10^1^**	
		W	16.3**		1.31 × 10^1^**	
		WS*W	5.94**		7.11 × 10^0^**	
		WS*W*T	32.7**		9.65 × 10^0^**	
	CV (%)		4.42		8.03	
	**Water source**		**Treated**	**Filtered**	**Treated**	**Filtered**
	Wastewater dose (%)	20	13.003 a	12.675a	9.275b	12.700^†^a
		40	14.923 a	13.500b	9.750b	13.375^†^a
		60	15.608 b	16.535a	10.725b	13.850^†^a
		80	12.003 ^†^b	14.488 a	13.475^†^b	15.210^†^a
		100	11.305 ^†^b	13.785 a	12.375^†^a	11.475a
	Control		10.785 ^†^		13.850 ^†^	

Cultivation cycles			Cycle 1		Cycle 2	
Shoot fresh mass (g pot^−1^)	Mean square	WS	2.70**		3.52 × 10^0^**	
		W	2.72**		1.06 × 10^0^**	
		WS*W	1.24**		6.90 × 10^−1^**	
		WS*W*T	4.77**		5.79 × 10^−2ns^	
	CV (%)		8.54		8.38	
	**Water source**		**Treated**	**Filtered**	**Treated**	**Filtered**
	Wastewater dose (%)	20	2.883a	2.945a	0.800b	1.365a
		40	3.273b	3.693a	0.990b	1.750^†^a
		60	3.585a	3.095b	1.173b	2.075a
		80	1.988^†^b	3.383a	1.505^†^b	2.635a
		100	1.410^†^b	2.623a	1.780^†^a	1.390b
	Control		1.743 ^†^		1.673 ^†^	

Cultivation cycles			Cycle 1		Cycle 2	
Shoot dry mass (g pot^−1^)	Mean square	WS	7.09 × 10^−3ns^		1.09 × 10^−1^**	
		W	1.74 × 10^−1^**		2.16 × 10^−2^**	
		WS*W	7.59 × 10^−2^**		3.20 × 10^−2^**	
		WS*W*T	2.24 × 10^−1^**		1.88 × 10^−3ns^	
	CV (%)		6.94		6.30	
	**Water source**		**Treated**	**Filtered**	**Treated**	**Filtered**
	Wastewater dose (%)	20	0.677a	0.576b	0.248b	0.305a
		40	0.793a	0.724b	0.263b	0.418^†^a
		60	0.932a	0.763b	0.270b	0.443a
		80	0.422^†^b	0.649a	0.305b	0.535a
		100	0.377^†^b	0.623a	0.415^†^a	0.323b
	Control		0.405 ^†^		0.374 ^†^	

Cultivation cycles			Cycle 1		Cycle 2	
Water productivity (kg m^−3^)	Mean square	WS	1.02 × 10^−4ns^		1.88 × 10^−3^**	
		W	2.57 × 10^−3^**		3.73 × 10^−4^**	
		WS*W	1.12 × 10^−3^**		5.51 × 10^−4^**	
		WS*W*T	3.31 × 10^−3^**		3.10 × 10^−5ns^	
	CV (%)		6.94		6.30	
	**Water source**		**Treated**	**Filtered**	**Treated**	**Filtered**
	Wastewater dose (%)	20	0.0822a	0.0699b	0.0325b	0.0400a
		40	0.0963a	0.0879b	0.0344b	0.0548^†^a
		60	0.1132a	0.0927b	0.0354b	0.0580a
		80	0.0512^†^b	0.0788a	0.0400b	0.0702a
		100	0.0458^†^b	0.0756a	0.0544^†^a	0.0423b
	Control		0.0493 ^†^		0.0490 ^†^	

*Note:* WS*W*T: interaction between water sources, wastewater proportions, and the control treatment; * and ** indicate significance at the 5% and 1% probability levels, respectively, by the *F*‐test; ns: not significant (*p* > 0.05); means followed by the same lowercase letter in the row do not differ between water sources within each crop cycle according to Tukey's test (*p* ≤ 0.05); means followed by † do not differ from the control according to Dunnett's test (*p* ≤ 0.05).

Abbreviation: CV: coefficient of variation.

Plant height showed clear differences between water sources and cultivation cycles. In the first cycle, filtered water showed superior performance compared with treated water, especially at the highest proportions, with greater values at 60% (16.54 cm), 80% (14.49 cm), and 100% (13.79 cm). However, at 40%, treated water resulted in greater plant height (14.92 cm), indicating that the response did not follow the same trend across the entire range of proportions. In the second cultivation cycle, the differences between water sources became more consistent. Plants irrigated with filtered WW showed greater plant height values at WW proportions ranging from 20% to 80%, varying from 12.70 to 15.21 cm, whereas treated water resulted in significantly shorter plants within this range, with values between 9.28 and 13.48 cm.

This behavior indicates that posttreatment with biochar contributed to mitigating limitations associated with water quality. Although the filtration system did not promote a consistent reduction in effluent salinity, a marked improvement in microbiological quality and dissolved oxygen was observed, factors that may have contributed to the better agronomic performance observed in part of the treatments (Çömez et al. 2024; Panghal et al. 2024; Shigei et al. 2024). This allowed the maintenance of vegetative growth even under high WW proportions. At the 100% proportion, however, the height of plants irrigated with filtered water decreased (11.48 cm), approaching the performance observed with treated water (12.38 cm) and remaining below the control treatment. This suggests that, at maximum effluent concentrations, the cumulative effects of management practices tend to reduce the benefits provided by the filtration system. Even so, the superior performance of filtered water across most evaluated proportions highlights greater maintained performance in plant height growth during the second cycle when compared with treated water alone.

SFM showed distinct responses between water sources and cultivation cycles. In the first cycle, WW filtered through green coconut biochar promoted greater SFM values across most evaluated proportions. The 40%, 80%, and 100% WW proportions were particularly notable, in which filtered water outperformed both treated water and the control treatment, indicating a positive effect of posttreatment on fresh biomass accumulation. In contrast, treated water resulted in a more pronounced reduction in SFM at the highest WW proportions, suggesting greater sensitivity of vegetative growth to increased effluent load when no additional filtration step was applied, a behavior frequently associated with increased osmotic stress and EC in systems irrigated with WW (Zhang et al. 2025).

In the second cycle, the observed pattern was similar to that identified for plant height. Filtered water maintained greater SFM values at WW proportions ranging from 20% to 80%, with production varying from 1.36 to 2.64 g pot^−1^, whereas treated water showed lower values within this range. This behavior indicates that the greater plant height observed under irrigation with filtered water was accompanied by greater fresh biomass accumulation, reflecting more favorable conditions for vegetative tissue expansion during the continued use of the effluent. At the 100% WW proportion, however, SFM under filtered water decreased, approaching the values observed for treated water and the control treatment, a pattern consistent with the reduction in plant height under this condition. These results suggest that although the biofilter mitigates the negative effects of WW reuse across most proportions, maximum WW concentrations may still limit vegetative growth even after posttreatment.

SDM showed responses consistent with the patterns observed for plant height and fresh mass, although with greater sensitivity to WW proportions. In the first cycle, treated WW promoted an increase in SDM up to the 60% WW proportion, indicating a positive effect of the nutrient supply provided by the effluent within this range. However, at the 80% and 100% proportions, a marked reduction in dry mass was observed under treated water, with values lower than those of the control treatment. In contrast, WW posttreated with green coconut biochar showed greater SDM values at these higher proportions, surpassing treated water and, in some cases, approaching or exceeding the control treatment. This behavior indicates that the biofilter contributed to mitigating limitations associated with increased effluent load, favoring structural biomass accumulation even under conditions with a greater proportion of WW, an effect also reported in recent studies involving the application of biochar in agricultural systems irrigated with restrictive‐quality water (Jiang et al. 2025). Similar results have been reported in recent studies involving biochar‐based biofilters applied to tertiary WW treatment, in which improvements in physicochemical and microbiological water quality resulted in greater productive performance of irrigated crops (Bui et al. 2024; Shigei et al. 2024; Wang et al. 2024).

In the second cycle, the difference between water sources became less pronounced, although the general pattern was maintained. Filtered water showed greater SDM values at the 20%, 40%, 60%, and 80% WW proportions, whereas treated water maintained lower values across these doses. At the 100% WW proportion, treated water showed a higher value than filtered water, indicating a possible limitation of the biofilter effect under continuous use and high effluent load. This result suggests that, over successive cycles, the gradual accumulation of salts in the soil may reduce the differential between water sources, although the filtration system still maintains advantages at intermediate proportions.

In addition, the second cycle was conducted under conditions of greater atmospheric demand, with mean solar radiation of 14.95 MJ m^−2^ day^−1^ and mean ETo of 3.27 mm day^−1^, compared with the first cycle (13.90 MJ m^−2^ day^−1^ and 2.95 mm day^−1^) (Figure 2). Although the differences were moderate, this scenario may have intensified the sensitivity of coriander plants to variations in water quality, especially at higher WW proportions, in which the balance between nutrient supply and osmotic stress tends to become more critical.

Overall, SDM reinforces the results observed for fresh mass. Whereas treated water showed responses that were more dependent on the applied proportion, with reductions at the highest doses, filtered water promoted greater consistency in dry matter accumulation, particularly at intermediate WW ranges. This pattern indicates that posttreatment with biochar favored not only vegetative tissue expansion but also the conversion of this growth into biomass effectively incorporated into the plant.

Water productivity clearly reflected the patterns observed for vegetative growth and coriander biomass accumulation, integrating the effects of water source and WW proportions across both cultivation cycles. In the first cycle, treated WW promoted an increase in WP up to the 60% WW proportion, followed by a marked reduction at the 80% and 100% doses. This behavior indicates that the nutrient supply from the effluent initially contributed to greater water use efficiency. However, the increase in saline load at higher proportions began to limit productive performance, reducing biomass return per unit of applied water. In contrast, WW posttreated with biochar maintained greater and more stable WP values at the 80% and 100% WW proportions, surpassing treated water and, in some cases, equaling or approaching the control treatment. This result is directly associated with the greater fresh and dry biomass accumulation observed under irrigation with filtered water at these proportions, indicating that the biofilter contributed to preserving water use efficiency even under greater participation of the effluent, a condition similar to that reported in recent studies involving agricultural biochar use aimed at improving crop water use and productive efficiency (Khairo et al. 2025). It should be noted that the WP values reported in this study were obtained under greenhouse conditions using individual plants cultivated in 6‐L pots, with WP calculated from the SFM of each plant and the total volume of water consumed during the cultivation cycle. Therefore, the absolute values should be interpreted within the context of this pot‐scale experimental system, and direct quantitative comparisons with field‐scale studies should be made with caution.

In the second cycle, water productivity showed less pronounced differences between water sources, reflecting the greater uniformity also observed for shoot biomass. Even so, filtered water maintained greater WP values across most evaluated proportions, whereas treated water showed lower performance, especially at intermediate doses. At the 100% WW proportion, both water sources showed values similar to the control treatment, suggesting that continuous effluent use reduced the contrast between treatments, possibly due to the gradual accumulation of salts in the soil. Recent studies highlight that the continuous use of WW may increase EC and soil salinization risk, especially in systems subjected to high ETc and successive cultivation cycles (Odone et al. 2024; Wang et al. 2024).

Taken together, the WP results confirm that water‐use efficiency was strongly associated with vegetative growth and biomass accumulation. Whereas treated water showed greater dependence on the applied proportion, with loss of efficiency at higher doses, the use of a green coconut biochar biofilter promoted greater productive stability, especially in the first cycle. This behavior reinforces the role of posttreatment as a strategy to reduce negative fluctuations in agronomic performance, particularly in agricultural reuse systems employing biochar‐based adsorbent materials to improve effluent quality (Marín et al. 2025). Furthermore, it contributes to increasing the predictability of agricultural reuse, especially under conditions with a greater proportion of WW.

Overall, the results indicate that WW posttreatment with green coconut biochar improved the agronomic performance of coriander. This effect was more evident during the first cultivation cycle and at intermediate WW proportions. Under these conditions, greater plant height, shoot biomass accumulation, and water productivity values were observed. These results indicate a better balance between the nutrient supply provided by the effluent and the mitigation of limiting effects associated with WW reuse. In the second cycle, the differences between water sources became less pronounced, suggesting the influence of continuous effluent use and gradual salt accumulation in the soil. Even so, filtered water maintained more stable responses across most evaluated proportions. This indicates that green coconut biochar contributed to reducing negative fluctuations in growth and water‐use efficiency, an important aspect for the feasibility of agricultural reuse under successive cultivation cycles.

Despite the positive results, the study was conducted under controlled conditions and with a limited number of cultivation cycles. This restricts inferences regarding the long‐term behavior of the system. The progressive accumulation of salts in the soil, the possible saturation of the biochar, and their effects on soil chemical and biological attributes were not continuously evaluated, although recent studies indicate that the prolonged use of carbonaceous materials in effluent‐irrigated systems may alter soil physicochemical properties and microbiological dynamics over cultivation cycles (Paliaga et al. 2025). Therefore, future studies should include larger scale experiments with a greater number of successive cultivation cycles, together with detailed monitoring of soil–water–plant interactions. In addition, engineering aspects related to the hydraulic loading capacity of the filtration system, the service life of the biochar, regeneration or replacement strategies, and the economic feasibility of large‐scale implementation should be evaluated to better define the operational limits and practical applicability of green coconut biochar as a supporting technology for agricultural WW reuse.

**FIGURE 7:**
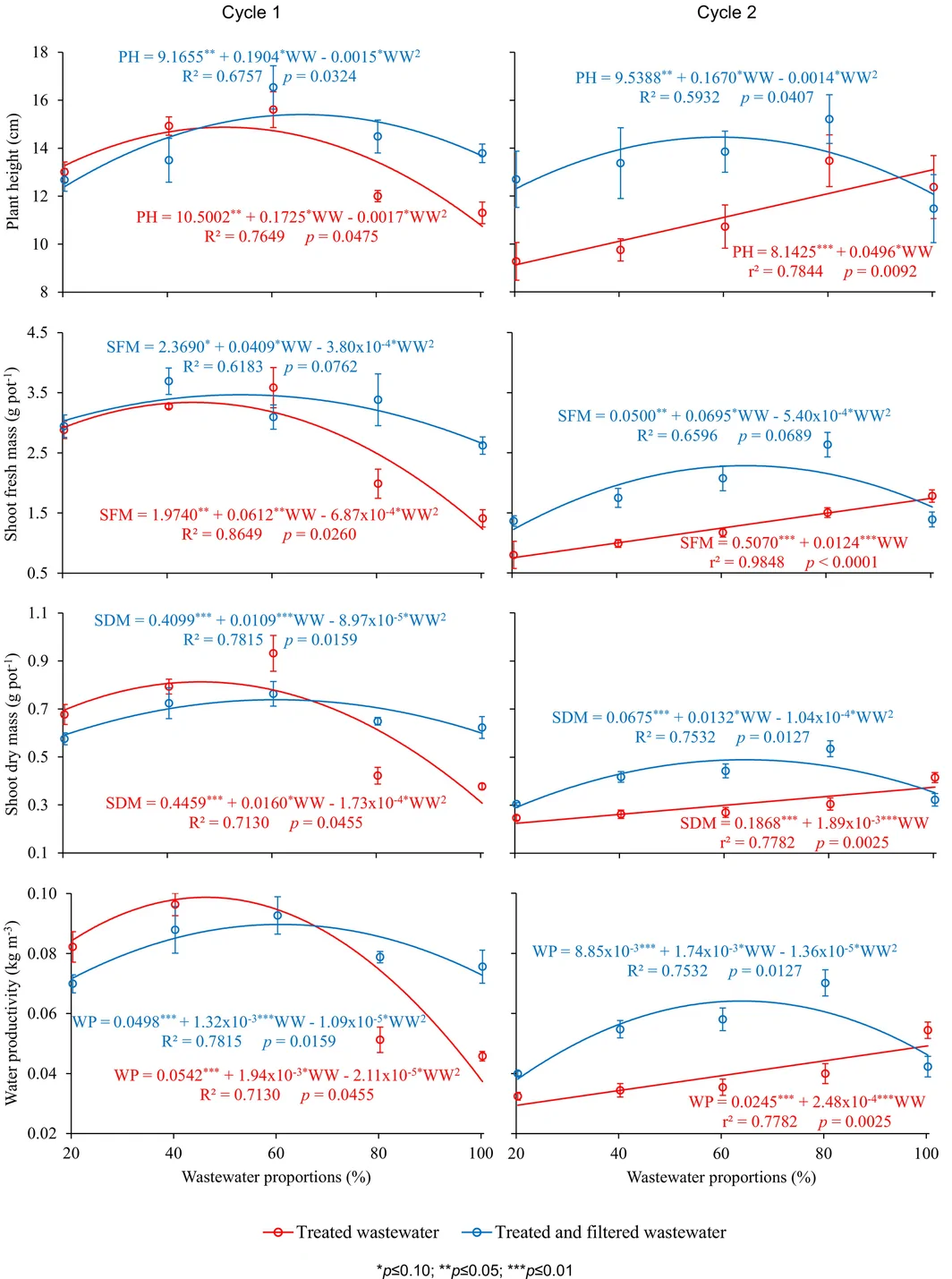
Plant height (PH), shoot fresh mass (SFM), shoot dry mass (SDM), and water productivity (WP) of coriander in two cultivation cycles as a function of wastewater (WW) proportions mixed with supply water, using either conventionally treated wastewater or wastewater additionally treated and filtered through a green coconut biochar biofilter.

Figure 7 presents the regression analysis results for coriander agronomic variables across two cultivation cycles as a function of WW proportions applied through fertigation. Conventionally treated WW and WW posttreated with green coconut biochar are compared, allowing the quantitative evaluation of crop responses to different effluent proportions, as well as changes in response patterns between cultivation cycles.

The quantitative response of coriander to WW proportions revealed distinct patterns between cycles and between the two water sources, highlighting the interaction between the nutrient supply from the effluent, the effect of accumulated salinity, and the efficiency of biochar posttreatment.

In the first cycle, plant height showed a quadratic behavior for both water sources. For conventionally treated WW, the maximum value was estimated at approximately 50.7% WW, resulting in a plant height of about 14.9 cm. For biochar‐filtered water, the peak occurred at a higher proportion, close to 63.5% WW, with an estimated height of 15.2 cm. This shift in the optimum point and the higher value observed with filtered water indicate that biochar contributed to modifying effluent composition, improving sanitary quality and dissolved oxygen availability, as well as favoring the retention of potentially harmful compounds to plant growth. Similar behavior has been reported in adsorption systems using biochars capable of removing salts, organic compounds, and light metals, resulting in improved water quality for agricultural purposes (Alshehri and Pugazhendhi 2024; Fang et al. 2025).

In the second cycle, the pattern changed, possibly associated with gradual salt accumulation in the soil after the first crop. This phenomenon has been documented in areas continuously irrigated with effluents, especially under hot climates and high evaporation rates (Marques et al. 2018; Silva et al. 2025; Perulli et al. 2026). Plants irrigated with conventionally treated WW showed a linear increasing response, reaching an estimated value of 13.1 cm at 100% WW, indicating that the effect of nutrient supply began to prevail over salt stress at high effluent levels. In contrast, filtered water maintained a quadratic response, with a maximum estimated at approximately 59.6% WW, resulting in a plant height of about 14.5 cm. Although the response amplitude was lower compared to the first cycle, filtered water maintained higher values across most evaluated proportions, suggesting a partial persistence of the beneficial effect of biochar, even under possible progressive saturation of the filtration material. This behavior has been reported in studies showing a gradual decline in biochar adsorption capacity over time (Bui et al. 2024; Ávila et al. 2023).

SFM followed a similar trend. In Cycle 1, both sources showed quadratic responses, with maximum values estimated at 44.5% WW for treated water (3.34 g pot^−1^) and 53.8% WW for filtered water (3.47 g pot^−1^). The higher value and the broader optimum range under filtered water indicate that biochar preserved the nutrient value of the effluent while also promoting more favorable conditions for plant growth even at high WW proportions, favoring cell expansion and fresh biomass accumulation. This mechanism has been observed in microfiltration and adsorption systems applied to agricultural reuse (Asirifi et al. 2023; Wang et al. 2024). In the second cycle, treated water showed a linear increasing response, reaching 2.23 g pot^−1^ at 100% WW, whereas filtered water maintained a quadratic response with a maximum estimated at approximately 64.4% WW, reaching 2.29 g pot^−1^. These results indicate that even after continuous effluent use, the biofilter maintained a more efficient response at intermediate proportions.

Dry shoot mass showed a pattern consistent with fresh mass but with greater sensitivity to WW proportions. In Cycle 1, treated WW showed a quadratic response, with a maximum at approximately 46.2% WW (0.82 g pot^−1^). For filtered water, the maximum occurred at a higher proportion, around 60.8% WW (0.74 g pot^−1^). Despite the higher value observed under treated water in Cycle 1, filtered water showed more stable performance at higher WW proportions. In Cycle 2, treated water showed a linear increase, reaching 0.38 g pot^−1^ at 100% WW, while filtered water maintained a quadratic response, with a maximum at approximately 63.5% WW (0.49 g pot^−1^). This result indicates that biochar contributed to greater structural biomass accumulation even under continuous WW use.

Water productivity synthesized the previously observed patterns. In Cycle 1, both sources showed quadratic responses, with maxima at 46.0% WW for treated water (0.099 kg m^−3^) and 60.6% WW for filtered water (0.090 kg m^−3^). Although treated water showed a slightly higher peak value in the first cycle, filtered water maintained greater and more consistent performance at higher WW proportions, indicating higher water use efficiency under greater effluent load. In Cycle 2, treated water showed a linear increase, reaching 0.049 kg m^−3^ at 100% WW, while filtered water showed a quadratic response, with a maximum at approximately 64.0% WW (0.064 kg m^−3^). These results confirm that biochar posttreatment improved water use efficiency, especially at intermediate WW proportions.

Overall, the results indicate that green coconut biochar posttreatment regulated coriander agronomic responses across cropping cycles. In the first cycle, the biofilter expanded the optimal range of WW proportions associated with maximum growth, biomass, and water productivity, indicating a better balance between nutrient supply and osmotic effects. In the second cycle, although the positive effect was partially reduced, filtered water still showed superior or more stable performance across most evaluated proportions, especially at intermediate levels. This behavior may be related to cumulative limitations associated with continuous WW use or a gradual reduction in biofilter efficiency over time. Nevertheless, the main benefit of biochar is not only limited to yield increases but also includes greater stability of agronomic responses under WW reuse systems.

However, the experiment was conducted under controlled conditions and over a limited number of cropping cycles, restricting long‐term inferences regarding WW reuse with biochar filtration. The lack of direct soil assessments and plant physiological measurements limits a more precise understanding of the mechanisms underlying the reduced responses in the second cycle. Therefore, future studies should include soil chemical and physical monitoring, longer successive cropping cycles, evaluation of biochar durability, regeneration or replacement strategies, hydraulic loading capacity, and the economic feasibility of large‐scale implementation, together with integrated soil–water–plant system analyses, to consolidate green coconut biochar as a sustainable technology for agricultural WW reuse. Green coconut biochar.

## 4 Conclusions

The use of domestic WW posttreated with green coconut biochar showed potential as a strategy for coriander fertigation, improving effluent quality and crop agronomic performance.

The filtration system was associated with increased dissolved oxygen and a significant reduction in microbiological load, as well as moderate reductions in organic load, while maintaining nutrient concentrations suitable for agricultural reuse. However, parameters such as EC and salinity were not significantly reduced, indicating that the biofilter primarily improves sanitary and physicochemical quality rather than removing dissolved salts.

From an agronomic perspective, filtered water provided better responses in plant height, biomass accumulation, and water productivity, especially at intermediate WW proportions. Biochar posttreatment also promoted more consistent productive responses, particularly in the first cropping cycle. In the second cycle, although the positive effect was reduced, filtered water still performed similarly or better than treated water under most conditions.

Regression analyses suggested that biochar use expanded the range of WW application associated with optimal crop performance, allowing higher WW reuse proportions without compromising coriander growth. However, continuous WW application suggests possible cumulative effects in the soil–plant system, mainly associated with salt accumulation.

Thus, green coconut biochar represents a promising alternative for WW posttreatment aimed at agricultural reuse, contributing to higher productivity efficiency and safer WW use. Nevertheless, long‐term studies are required to evaluate filter durability, hydraulic loading capacity, biochar regeneration or replacement strategies, soil salt accumulation, and the economic feasibility of large‐scale implementation.

## Author Contributions

**Antonio Magno dos Santos Souza:** conceptualization, formal analysis, investigation, methodology, project administration, validation, visualization, writing – original draft, writing – review and editing, data curation. **Fernando França da Cunha:** formal analysis, methodology, resources, writing – review and editing, conceptualization, funding acquisition, supervision, project administration, validation, visualization. **Fabiano Santos Santana:** investigation, data curation. **Gregório Guirado Faccioli:** investigation, resources, writing – review and editing. **Luciano de Melo:** investigation, resources, writing – review and editing. **Nicole Helen Lima Cardeal:** data curation, investigation.

## Funding

This research was funded by the Coordination for the Improvement of Higher Education Personnel, Brazil (CAPES), the Finance Code 001, and the National Council for Scientific and Technological Development, Brazil (CNPq), Process 308769/2022‐8.

## Conflicts of Interest

The authors declare no conflicts of interest.

## Data Availability

All relevant data are included in the paper or its Supporting Information.
